# Altered light-dark phase-dependent behavioral responses and suprachiasmatic nucleus pathology in an *α*-synuclein rat model of Parkinson’s disease

**DOI:** 10.1038/s41531-026-01436-2

**Published:** 2026-06-19

**Authors:** Hanna Weber, Meike Statz, Nicolas Casadei, Olaf Riess, Franziska Richter, Wiebke Hermann, Alexander Storch, Mareike Fauser

**Affiliations:** 1 Department of Neurology, University Medical Center Rostock, University of Rostock, 18147 Rostock, Germany; 2 Institute of Medical Genetics and Applied Genomics, University of Tübingen, 72076 Tübingen, Germany; 3 Center for Rare Diseases, University of Tübingen, 72076 Tübingen, Germany; 4 Center for Systems Neuroscience Hannover (ZSN), 30559 Hannover, Germany; 5 Department of Pharmacology, Toxicology and Pharmacy, University of Veterinary Medicine Hannover, 30559 Hannover, Germany; 6 German Centre for Neurodegenerative Diseases (DZNE) Rostock-Greifswald, 18147 Rostock, Germany

**Keywords:** Neuroscience, Physiology

## Abstract

Disruption of circadian rhythms is a key feature of neurodegenerative diseases and a non-motor feature of Parkinson’s disease, which significantly impairs health-related quality of life; yet, the underlying mechanisms are only partially understood. Preclinical animal models with neuropathological and symptomatic significance might contribute to a better understanding of circadian dysfunction. Here, we investigated light-dark phase-dependent modulation of motor and non-motor behavior as well as suprachiasmatic nucleus integrity in *α*-synuclein-overexpressing rats and wild-type controls. Behavioral testing in 3-month-old animals revealed robust phase-dependent modulation of exploratory activity, locomotion, sucrose preference, and olfaction-guided feeding in wild-type rats, which was absent in their transgenic littermates. Histological analyses demonstrated reduced overall cell density and pronounced *α*-synuclein accumulation in the suprachiasmatic nucleus of *α*-synuclein rats, accompanied by altered cellular composition, including altered neuronal, orexinergic, and microglial markers. *α*-synuclein load was positively correlated with Orexin A^+^ fibers and Iba1^+^ cell counts, suggesting a link between protein aggregation, neuroinflammation, and altered light-dark phase-dependent behavior. These findings indicate that *α*-synuclein rats lack phase-dependent behavioral alternations and exhibit suprachiasmatic nucleus pathology already at an early disease stage with very mild motor impairment, providing a translational model to study different aspects of non-motor symptoms in Parkinson’s disease.

## Introduction

Parkinson’s disease (PD) is a progressive neurodegenerative disorder, which is characterized by motor symptoms like bradykinesia, tremor, rigidity, and postural instability^1^. Motor dysfunction is accompanied by a degeneration of dopaminergic neurons in the midbrain^2,3^, progressive accumulation of *α*-synuclein^4,5^, and activation of microglia causing neuroinflammation^6,7^. Traditionally, PD has been diagnosed based solely on motor symptoms; recent research has increasingly recognized the significance of non-motor symptoms, such as hyposmia, anxiety, depression, and anhedonia, which greatly impact health-related quality of life in PD patients^8–10^.

Already in James Parkinson’s first description of the disease in 1817, he reported that patients frequently suffered from sleep-associated symptoms, for example, daytime sleepiness and altered sleep architecture^11^. Today, it is understood that sleep disturbances represent only one aspect of a broader circadian dysfunction affecting PD patients, including hormonal dysregulation, particularly of cortisol and melatonin, and related autonomic dysfunction^12–15^. These circadian alterations are likely triggered by neurophysiological and neuropathological changes, potentially involving epigenetic modifications of clock genes, neuroinflammation, and structural alterations within the suprachiasmatic nucleus (SCN), the central circadian pacemaker^15,16^.

However, some pathophysiological mechanisms are challenging to study in humans, highlighting the importance of animal models. Despite this need, only a limited number of studies in PD animal models have addressed circadian rhythmicity beyond sleep.

Most investigations in toxin-based models (6-hydroxydopamine (6-OHDA), 1-methyl-4-phenyl-1,2,3,6-tetrahydropyridine (MPTP), rotenone) in mice or rats have focused on the expression of clock genes, altered sleep architecture, or general locomotor activity^17–21^. These studies consistently report that wild-type (WT) animals show robust phase-dependent differences in motor performance, while PD model animals lack such modulation, suggesting a disruption of day/night rhythmicity in these disease models. In genetic PD models, research has similarly emphasized sleep disruption^22–24^, with only a single study assessing motor activity and reporting circadian impairment^25^. Several studies in genetic mouse models demonstrated *α*-synuclein deposition in the SCN, accompanied by structural and functional alterations^25–27^. Thus, pathology in the SCN may contribute to altered behavioral responses across circadian phases, which in turn contribute to a range of early non-motor symptoms of PD.

To address this hypothesis, we investigated both behavioral domains associated with non-motor symptoms of PD and histological alterations within the SCN. For this purpose, we employed a genetic PD rat model that expresses the full-length wild-type human *α*-synuclein under a human promoter (SNCA rats)^28^. This model recapitulates key aspects of PD pathology, including progressive *α*-synuclein accumulation and age-dependent dopaminergic degeneration, and also shows early non-motor phenotypes such as reduced novelty-seeking, avoidance behavior, and impaired olfaction, even before overt motor deficits emerge. For our study, we chose young rats aged three months with a phenotype resembling early-stage PD, including the aforementioned early non-motor symptoms without overt motor pathology^29,30^.

In this study, we specifically investigated the impact of light phase on behavioral performance in WT and SNCA rats. We hypothesized that SNCA rats would exhibit impaired light-dark phase-dependent behavioral modulation, in particular related to motivation, motor performance, and anxiety. To test this hypothesis, animals were divided into two experimental cohorts based on light phase: the active group (tested during the dark phase of their day/night and the inactive group (tested during the light phase). A series of behavioral assays, including the light-dark box test (LDB) to assess anxiety and exploratory behavior, the buried pellet test (BPT) and surface pellet test (SFPT) to evaluate motivational and olfactory function, and the sucrose preference test (SPT) to measure (an)hedonic behavior, were performed.

Beyond behavioral outcomes, we also examined the cellular composition of the SCN, with a particular focus on the neuropeptides vasoactive intestinal peptide (VIP), vasopressin (VP), and Orexin A (also known as hypocretin). While VIP^31,32^ and VP^33^ represent the two major neuropeptidergic subpopulations within the SCN, mediating synchronization of cellular oscillators and contributing to the generation and stability of circadian output signals, respectively^31–33^, Orexin is predominantly expressed in lateral hypothalamic neurons and plays a key role in regulating arousal, appetite, energy homeostasis, stress responses, visceral function, and wakefulness^34–36^.

Given the known accumulation of *α*-synuclein and neuroinflammatory processes in PD, we assessed whether SNCA rats show corresponding histological alterations in the SCN. Our findings reveal a marked difference in light–dark phase-dependent behavioral modulation between WT and SNCA rats, accompanied by reduced overall cell density, *α*-synuclein load, and shifts in specific neuronal and glial populations within the SCN. Together, these results suggest that the SNCA model exhibits impaired light-dark phase-dependent behavior at both the behavioral and cellular levels, with potential implications for understanding the link between early emerging non-motor symptoms and PD progression.

## Results

### Young SNCA rats show light–dark phase-dependent behavioral pathology

**Fig. 1:**
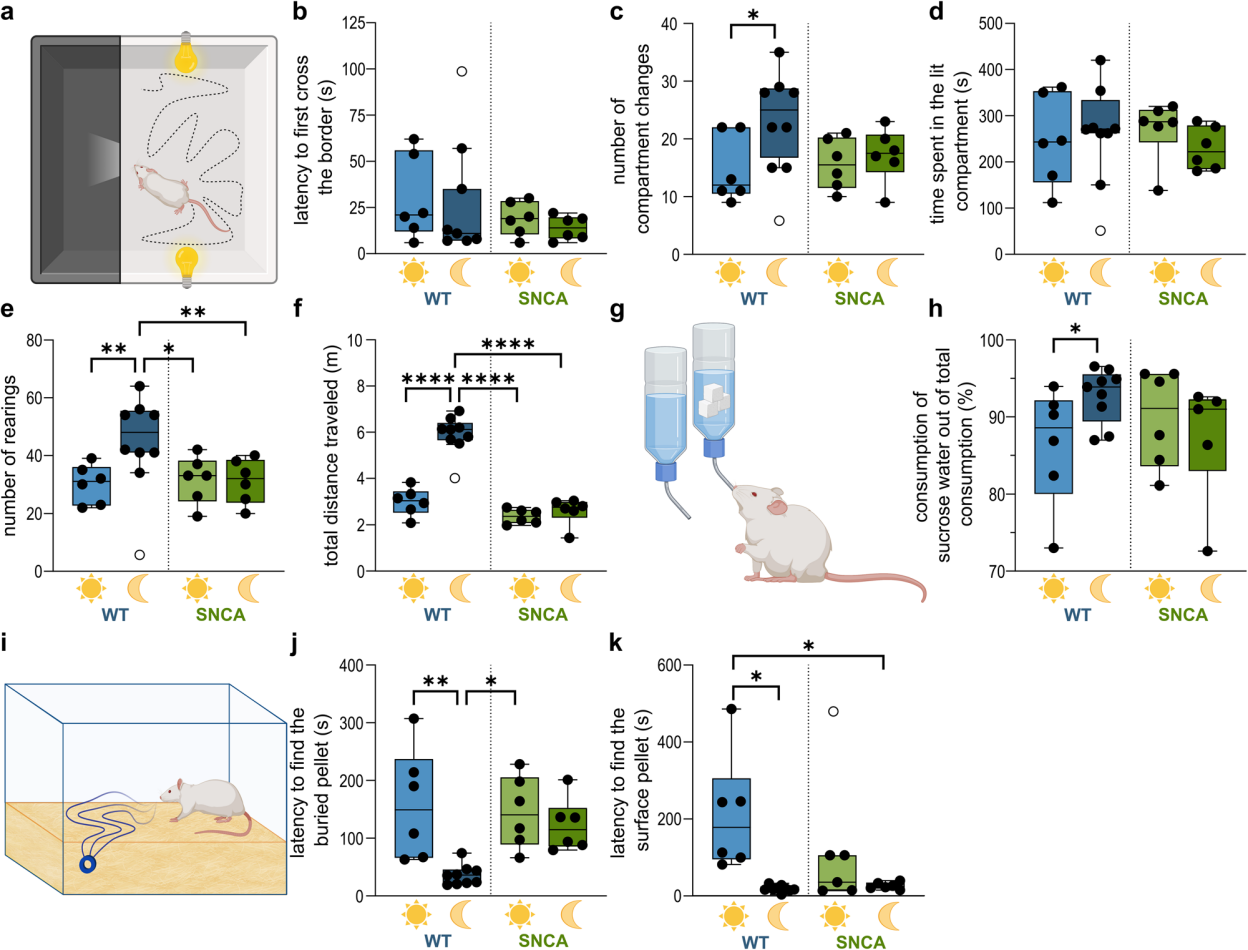
Light–dark phase-dependent modulation of behavioral performance in wild-type (WT) and *α*-synuclein overexpressing rats (SNCA rats). **a** Schematic illustration of the light–dark box (LDB) test. **b** Latency to the first transition between compartments in the LDB did not differ between groups or the light (sun) and dark (moon) phases. **c** Number of compartment changes in the LDB, which was significantly increased in WT animals tested during their active phase compared to their inactive phase, whereas no light-dark phase-dependent modulation was observed in SNCA rats. **d** Time spent in the light compartment of the LDB did not vary across genotypes or phases. **e** Number of rearing events in the LDB, with WT animals showing increased rearing during the active phase compared to the inactive phase; rearing counts were also higher in active WT than in SNCA rats under both light conditions. **f** Total distance traveled in the LDB, following the same pattern as rearing behavior (WT active > WT inactive; WT active > SNCA active and inactive). **g** Schematic illustration of the sucrose preference test (SPT). **h** Percentage of sucrose solution consumed relative to total fluid intake, which increased in WT rats during the active phase, whereas no phase-dependent modulation was detected in SNCA rats. **i** Schematic illustration of the buried pellet test (BPT). **j** Latency to retrieve a buried food pellet in the BPT, with WT rats being faster in the active compared to the inactive phase, and WT animals in the active phase performing significantly better than SNCA animals in the inactive phase. **k** Latency to retrieve a visible food pellet in the surface pellet test (SFPT), which was reduced in WT rats during the active compared to the inactive phase; additionally, WT animals tested during the inactive phase required significantly longer than SNCA animals tested during the active phase (one outlier from the SNCA inactive group outside of the plotted scale). Symbols indicate light phase (moon = active, sun = inactive). Data are presented as boxplots with individual data points overlaid. Asterisks indicate significance levels determined by two-way ANOVA, followed by Bonferroni’s multiple comparisons test (^*^p < 0.05, ^**^p < 0.01, ^***^p < 0.001, ^****^p < 0.0001). Partially created in BioRender. Weber, H. (2025) https://BioRender.com/tvf158v.

To investigate the effects of light–dark phases and genotype on behavioral performance, we conducted a series of tests assessing exploratory behavior, anxiety, motivation, olfaction, and anhedonia in WT and SNCA transgenic rats. Animals were tested either during their active (dark) or inactive (light) phase, as shown in Fig. 1.

In the LDB test, no significant differences were observed in the latency to first transition into the dark compartment (*F*(3, 14) = 1.112; *p* = 0.3775; Fig. 1b) or in the time spent in the light compartment (*F*(3, 14) = 0.8441; *p* = 0.4923; Fig. 1d). However, the number of compartment changes was significantly altered (*F*(3, 14) = 3.922; *p* = 0.0318; Fig. 1c). More precisely, we found significantly higher numbers in WT rats during the active phase compared to the inactive phase (*p* = 0.0401), in contrast SNCA rats showed no light–dark phase-dependent modulation. Exploratory activity, measured as rearing events, revealed robust phase-dependent modulation in WT animals (*F*(3, 14) = 7.336; *p* = 0.0034; Fig. 1e). WT rats displayed significantly more rearings during the active compared to the inactive phase (*p* = 0.0064). WT animals tested in their active phase additionally outperformed SNCA rats in the active (*p* = 0.0097) and inactive phase (*p* = 0.0119). In SNCA rats, no phase-dependent differences were detected. Locomotor activity followed the same pattern (*F*(3, 14) = 63.73; *p* < 0.0001; Fig. 1f): WT animals traveled significantly greater distances during the active compared to the inactive phase (*p* < 0.0001) and were more active than SNCA rats in their active (*p* < 0.0001) and their inactive phase *p* < 0.0001).

These findings suggest that light–dark phase-dependent behavioral differences in WT rats extend beyond basic activity levels, potentially influencing motivational or reward-related behaviors. Sucrose preference, expressed as the proportion of sucrose intake relative to total fluid consumption, revealed significant light–dark phase-dependent modulation in the WT but not in the SNCA rats (*F*(3, 92) = 2.966; *p* = 0.0361; Fig. 1h). WT rats during their active phase had an increased percentual intake of sucrose water compared to their inactive phase (*p* = 0.0395). In contrast, SNCA rats showed no light–dark phase-dependent modulation of sucrose preference.

To further investigate whether such light–dark phase-dependent effects generalize to olfactory-guided foraging, we next assessed performance in the buried and surface food pellet tests. In the BPT, WT rats again demonstrated clear light–dark phase-dependent modulation (*F*(3, 15) = 6.829; *p* = 0.0040; Fig. 1j), retrieving buried pellets significantly faster during the active compared to the inactive phase (*p* = 0.0057). Additionally, WT rats in the active phase outperformed SNCA animals tested during the inactive phase (*p* = 0.0136). In SNCA rats, retrieval latencies did not differ between phases. In the SFPT, again, light–dark phase-dependent modulation was only detected in WT rats (*F*(3, 14) = 6.429; *p* = 0.0058; Fig. 1k). WT rats retrieved the visible pellet significantly faster during the active compared to the inactive phase (*p* = 0.0117). Moreover, retrieval latencies were longer in WT animals tested during the inactive phase than in SNCA animals tested during the active phase (*p* = 0.0156).

### Three-month-old SNCA rats show no signs of dopaminergic degeneration

As expected, we found no dopaminergic degeneration in the SN (TH^+^_WT_ = 19,013 ± 7340 N/mm^2^; TH^+^_SNCA_ = 16,744 ± 8861 N/mm^2^; unpaired *t* test, *p* = 0.4905; Supplementary Fig. S2d), and VTA (TH^+^_WT_ = 24,763 ± 6771 N/mm^2^; TH^+^_SNCA_ = 21,903 ± 8710 N/mm^2^; unpaired *t* test, *p* = 0.3646; Supplementary Fig. S2f). Also, the fiber density in the dorsal striatum (TH^+^ fibers_WT_ = 40.74 ± 18.75; TH^+^ fibers_SNCA_ = 29.56 ± 13.18; Welch’s *t* test, *p* = 0.0939; Supplementary Fig. S2e) and the ventral striatum (TH^+^ fibers_WT_ = 37.52 ± 16.28; TH^+^ fibers_SNCA_ = 30.70 ± 15.21; unpaired *t* test, *p* = 0.2958; Supplementary Fig. S2g) was unaltered. As reported in the literature, a significant degeneration would be expected as late as around the age of 10–12 months^30,37^.

### *α*-synuclein accumulation associates with shifts in SCN cell populations

**Fig. 2:**
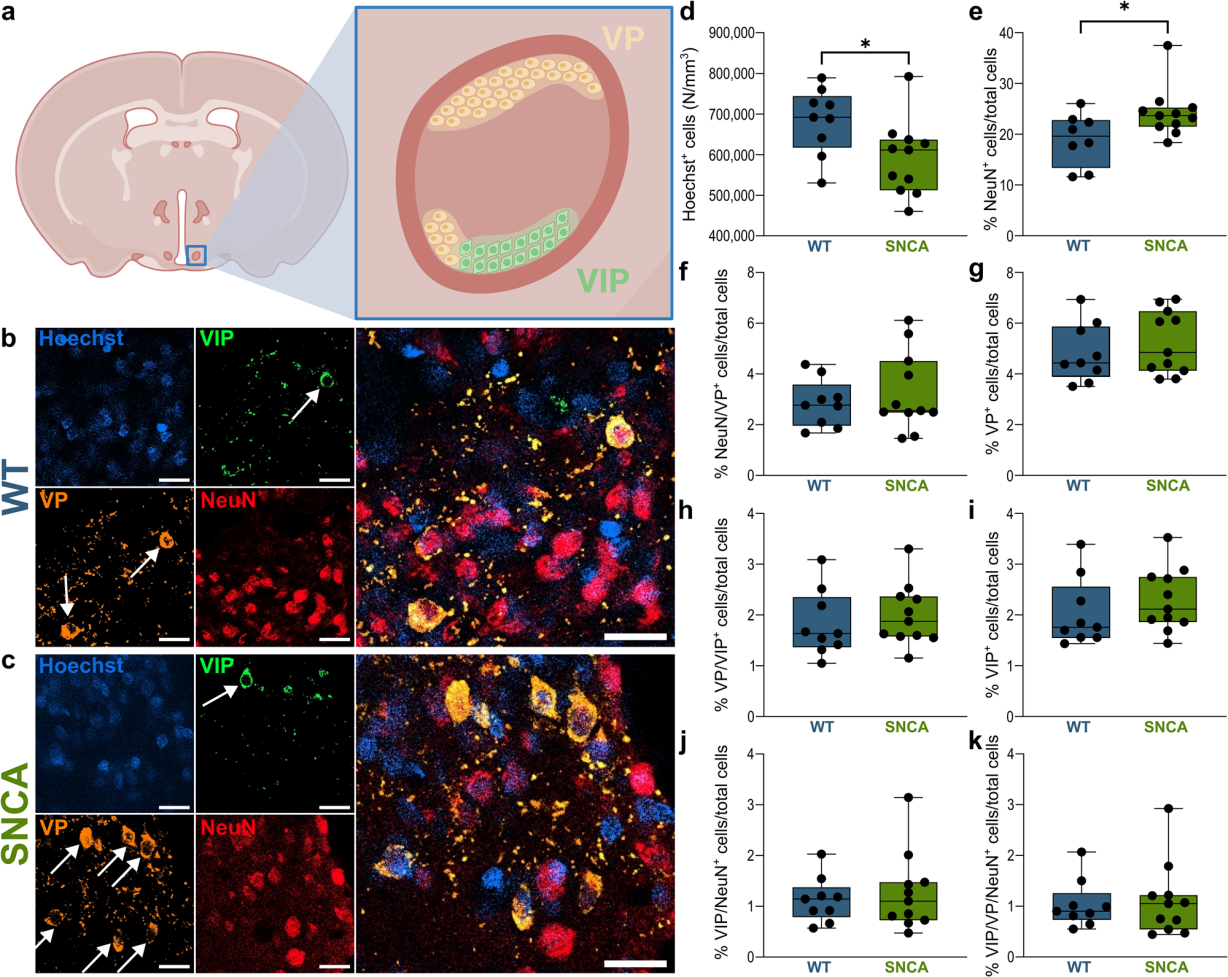
Histological characterization of the suprachiasmatic nucleus (SCN) in wild-type (WT) and *α*-synuclein overexpressing rats (SNCA rats) with focus on vasopressin (VP), vasoactive intestinal peptide (VIP), and NeuN positive cells. **a** Schematic illustration of a coronal brain section with magnification of the SCN, indicating the distribution of VIP^+^ and VP^+^ cells. **b** Representative histological staining of the SCN in WT rats. **c** Representative SCN staining from SNCA rats. **d** Quantification of total cell number via Hoechst staining in the SCN revealed significantly lower total cell counts in SNCA rats compared to WT. **e** The proportion of NeuN^+^ cells relative to total nuclei was elevated in SNCA rats compared to WT. **f**–**k** Relative proportions of NeuN^+^/VP^+^, VP^+^, VP^+^/VIP^+^, VIP^+^, VIP^+^/NeuN^+^, and VIP^+^/VP^+^/NeuN^+^ cells, expressed as percentages of the total Hoechst^+^ population. No significant differences were detected between those groups. Data are presented as boxplots with individual data points overlaid. Asterisks indicate significance levels determined by unpaired *t* tests (*p < 0.05). Scale bar = 20 μm. Partially created in BioRender. Weber, H. (2025) https://BioRender.com/5262s0p.

To investigate whether the observed behavioral alterations are mirrored by light–dark phase-dependent changes in regulatory structures, we next examined the cellular composition of the SCN (Fig. 2).

Histological examination of the SCN revealed group differences in both overall and relative cell counts. The total number of Hoechst^+^ nuclei was significantly reduced in SNCA rats compared to WT (cell counts_WT_ = 683,035 ± 81,772 N/mm^3^; cell counts_SNCA_ = 590,702 ± 91,46 7N/mm^3^; unpaired *t* test, *p* = 0.0302; Fig. 2d). To account for this reduction in total SCN cell density, all subsequent analyses of specific markers were normalized to the total Hoechst^+^ population. Therefore, the reported values represent the percentage of SCN cells expressing each marker, providing a relative measure of cell type composition within the SCN. Analysis of relative cell composition showed that the proportion of NeuN^+^ cells was significantly higher in SNCA rats (% NeuN^+^_WT_ = 18.99 ± 5.16%; % NeuN^+^_SNCA_ = 24.27 ± 4.96%; Welch’s *t* test, *p* = 0.0411; Fig. 2e). No significant differences were observed in the relative proportions of NeuN/VP^+^ (% NeuN/VP^+^_WT_ = 2.85 ± 0.93%; % NeuN/VP^+^_SNCA_ = 3.27 ± 1.56%; unpaired *t* test, *p* = 0.4840), VP^+^ (% VP^+^_WT_ = 4.84 ± 1.15%; % VP^+^_SNCA_ = 5.24 ± 1.25%; unpaired *t* test, *p* = 0.4605), VP/VIP^+^ (% VP/VIP^+^_WT_ = 1.82 ± 0.65%; % VP/VIP^+^_SNCA_ = 2.00 ± 0.60%; unpaired *t* test, *p* = 0.5456), VIP^+^ (% VIP^+^_WT_ = 2.04 ± 0.67%; % VIP^+^_SNCA_ = 2.29 ± 0.62%; Welch’s *t* test, *p* = 0.3940), VIP/NeuN^+^ (% VIP/NeuN^+^_WT_ = 1.13 ± 0.45%; % VIP/NeuN^+^_SNCA_ = 1.27 ± 0.76%; Welch’s *t* test, *p* = 0.6163), or VIP/VP/NeuN^+^ (% VIP/VP/NeuN^+^_WT_ = 1.04 ± 0.47%; % VIP/VP/NeuN^+^_SNCA_ = 1.11 ± 0.72%; Welch’s *t* test, *p* = 0.7968) cells between WT and SNCA rats (Fig. 2f–k).

**Fig. 3:**
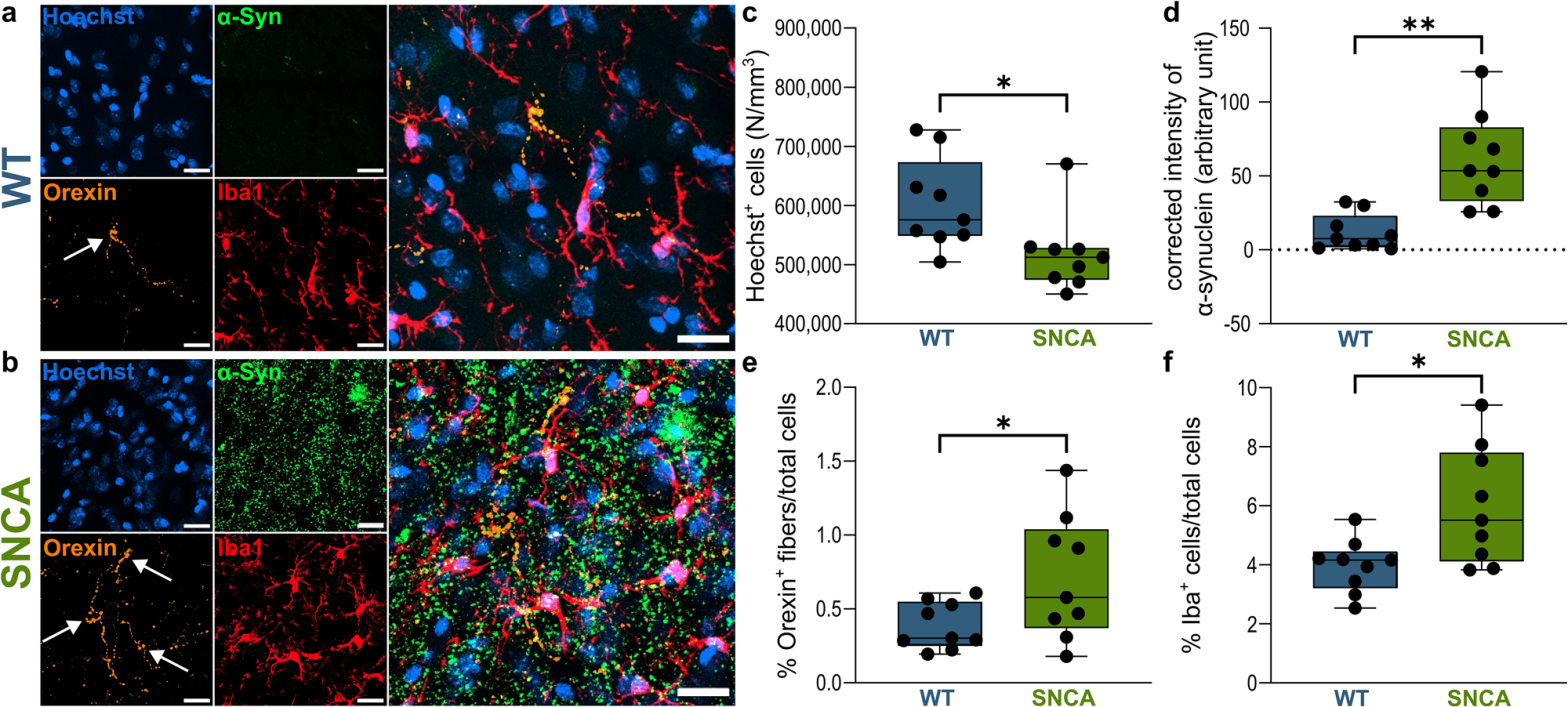
Altered cellular composition and *α*-synuclein accumulation in the suprachiasmatic nucleus (SCN) of *α*-synuclein overexpressing rats (SNCA rats). **a** Representative histological staining of the SCN in wild-type (WT) rats. **b** Corresponding staining in SNCA rats. **c** Quantification of total cell counts (Hoechst nuclei) within the SCN revealed significantly fewer cells in SNCA rats compared to WT. **d** Intensity analysis of *α*-synuclein immunoreactivity demonstrated markedly higher *α*-synuclein levels in SNCA rats, consistent with the human *α*-synuclein transgene expression being absent in WT animals. **e** Proportion of Orexin A^+^ fibers relative to total nuclei, showing increased percentages in SNCA compared to WT. **f** Proportion of Iba1^+^ cells relative to total nuclei, also elevated in SNCA rats compared to WT. Data are presented as boxplots with individual data points overlaid. Asterisks indicate significance levels determined by an unpaired *t* test (*p < 0.05, **p < 0.01). Scale bar = 20 μm.

To further characterize the cellular underpinnings of these alterations, we extended our analysis of the SCN to additional markers, including *α*-synuclein, Orexin A, and Iba1, to assess potential changes in neuropathology and hypothalamic projections. Histological analysis revealed distinct cellular alterations between WT and SNCA rats Fig. 3. Quantification of total Hoechst^+^ nuclei demonstrated again a significant reduction in overall cell numbers in SNCA compared to WT animals (cell counts_WT_ = 602,761 ± 77,238 N/mm^3^; cell counts_SNCA_ = 517,840 ± 63,586 N/mm^3^; Welch’s *t* test, *p* = 0.0220; Fig. 3c). As expected, *α*-synuclein immunoreactivity was strongly elevated in the SCN of SNCA rats, whereas WT animals showed no detectable staining (corrected *α*-synuclein intensity _WT_ = 11.41 ± 11.46; corrected *α*-synuclein intensity_SNCA_ = 61.47 ± 31.02; Welch’s *t* test, *p* = 0.0010; Fig. 3d). The relative proportion of Orexin A^+^ fibers was significantly higher in SNCA rats (% Orexin A^+^_WT_ = 0.39 ± 0.16%; % Orexin A^+^_SNCA_ = 0.71 ± 0.42%; unpaired *t* test, *p* = 0.0440; Fig. 3e). Similarly, the percentage of Iba1^+^ cells was increased in SNCA animals compared to WT (% Iba1^+^_WT_ = 3.97 ± 0.90%; % Iba1^+^_SNCA_ = 5.99 ± 2.00%; unpaired *t* test, *p* = 0.0440; Fig. 3f).

### *α*-synuclein accumulation aligns with Iba1^+^ cell and Orexin A^+^ fiber counts in SNCA rats

Correlation analyses were conducted to investigate the relationship between *α*-synuclein immunoreactivity and cellular composition within the SCN. Corrected *α*-synuclein intensity values were correlated with total Hoechst^+^ cell counts as well as the relative proportions of NeuN^+^, VP^+^, VIP^+^, Orexin A^+^, and Iba1^+^ cells or fibers using Pearson correlation tests. The corresponding heatmap can be found in the Supplementary Fig. S3. In WT rats, no significant correlations were observed, consistent with the absence of detectable *α*-synuclein staining. In contrast, SNCA rats exhibited uniformly positive correlations between *α*-synuclein intensity and all examined cellular parameters, with the associations for Iba1^+^ cells (*r* = 0.7135, *p* = 0.0205) and Orexin A^+^ fiber counts (*r* = 0.7748, *p* = 0.0085) reaching statistical significance. These results suggest that higher *α*-synuclein accumulation in the SCN of SNCA rats might be associated with increased proportions of Iba1^+^ microglia and Orexin A^+^ fibers.

## Discussion

In this study, we investigated whether impaired light-dark phase-dependent modulation emerges during the earliest stages of PD pathology in a genetic SNCA rat model. By combining behavioral testing across light phases with histological analyses of the SCN, we aimed to investigate how light–dark phase-dependent behavioral changes relate to underlying molecular and cellular alterations. Our results reveal that even in the absence of overt dopaminergic neurodegeneration, SNCA rats display impaired light-dark phase-dependent modulation of behavior, accompanied by *α*-synuclein accumulation, neuroinflammation, and altered orexinergic input within the SCN.

Patients with early-stage PD already suffer from a range of non-motor symptoms associated with sleep and circadian rhythm, including disturbances of the sleep–wake cycle, sleep fragmentation, excessive daytime sleepiness, rapid eye movement sleep behavior disorder (RBD), and restless legs syndrome (RLS)^38–40^. Some of these non-motor symptoms, such as RBD, manifest even before the onset of motor impairments, known as prodromal PD^41–44^. Other studies have reported altered rest-activity patterns in early PD patients^45,46^.

Alike, our animal model represents an early stage of the disease, without dopaminergic degeneration in the SN and the VTA. In the literature, dopaminergic cell loss in this model was shown to occur at the age of ten to twelve months^30,37^, and our own, so far unpublished data, indicate a midbrain neuronal loss of around 25% in the SN at ten to 12 months of age. Besides the absence of frank dopaminergic degeneration, our three-month-old SNCA rats only displayed minor behavioral differences compared to WT animals. These genotype-specific differences were limited to motor-related parameters, namely the total distance traveled and the number of rearing events in the LDB test. Therefore, our data clearly indicate that the animals already exhibit differences in light–dark phase-dependent behavior: while WT rats displayed clear behavioral phase-dependent modulation, SNCA animals showed no such variations. This strongly suggests that altered light–dark phase-dependent behavior occurs very early in the course of the disease and prior to the majority of other symptoms.

Consistent with our findings, the phenomenon of altered light-dark phase-dependent locomotor parameters has been investigated in various rodent models of PD^18,20,21,47–50^. In these toxin-based models, disrupted light–dark phase-dependent locomotor rhythms have often been attributed to a significant loss of dopaminergic neurons in the SN^48,50^ or the VTA^51^. In contrast, we used young animals (3 months of age) without signs of midbrain dopaminergic degeneration, yet altered light-dark phase-dependent behavior was still present. However, our study does not account for alterations that precede frank cellular degeneration, such as changes in dopaminergic plasticity or related mechanisms. Interestingly, recent studies suggest that not only dopaminergic degeneration, but also an altered distribution and activity of dopamine receptor subtypes, which have been described in both humans^52^ and genetic^53–55^, and non-genetic^56^ PD animal models, may contribute to circadian dysregulation. D1- and D2-like receptors are differentially expressed within the SCN and other circadian-relevant brain regions, where they modulate neuronal excitability and entrainment processes^56–58^. Subtle alterations in receptor signaling—even in the absence of measurable cell loss—could therefore interfere with the fine-tuned dopaminergic modulation of light-dark phase-dependent behavior. This provides a potential mechanistic explanation for why young SNCA rats, which have not yet developed midbrain dopaminergic neurodegeneration, already exhibit light-dark phase-dependent behavior disturbances.

Consistent with this, it is noteworthy that the original description of these SNCA rats by Nuber and colleagues also included an analysis of light–dark phase-dependent rhythmicity over a continuous 72 h period at different age groups. In this study, no significant differences in phase-dependent activity were reported at 6, 12, or 16 months of age^28^. At first glance, these results appear to contrast our findings of impaired light-dark phase-dependent behavior already at three months of age. However, the discrepancy can likely be explained by methodological differences: while Nuber et al. monitored gross locomotor rhythms in a familiar environment, our study specifically compared behavioral performance across light phases in dedicated test paradigms. Such task-related measures may be more sensitive in detecting subtle light-dark phase-dependent behavior impairments that remain undetectable in spontaneous activity recordings. Our results, therefore, complement and extend the original characterization of this model, revealing early light–dark phase-dependent behavior alternations that become evident only under phase-specific behavioral testing.

Behavioral testing was intentionally performed under the respective light-dark conditions (light during the inactive phase and darkness during the active phase). Under these conditions, photic masking, defined as the direct response to light independent of endogenous rhythmicity, represents a physiological mechanism that interacts with the circadian system to shape behavioral output^59,60^. In nocturnal species, light suppresses activity whereas darkness promotes it, thereby reinforcing light–dark phase-dependent behavioral patterns^61,62^. Importantly, for the SPT and the BPT, experimental illumination was aligned with the respective circadian phase, with light during the inactive phase and darkness during the active phase. This approach avoided introducing conflicting photic masking effects that could differentially influence behavioral outcomes between phases. In contrast, the LDB inherently includes both illuminated and dark compartments, irrespective of the external lighting condition. As a result, animals are exposed to conflicting photic cues in both phases, which may induce masking effects within this specific paradigm. Given the experimental design, it is not possible to disentangle the extent to which photic masking, as opposed to phase-dependent regulation, contributes to the observed behavior in the LDB.

Given that photic masking is mediated by specific retinal pathways, it is relevant to consider the underlying neurobiological mechanisms. Retinal intrinsically photosensitive ganglion cells (ipRGCs), which express melanopsin, mediate both circadian entrainment to light and photic masking by transmitting light information directly to the SCN and subcortical structures^63–65^. Notably, studies in PD patients suggest that ipRGC/melanopsin-mediated signaling can be impaired. Patients show reduced melanopsin-driven pupillary responses, correlating with sleep and circadian disturbances, even when outer retinal photoreceptors are intact^66,67^. These findings indicate that light signal transmission via ipRGCs may be compromised in PD, potentially affecting photic masking and light–dark phase-dependent regulation. To our knowledge, the literature does not provide data on the integrity of the retina and the entire visual system in our animal model.

Consequently, the pronounced light-dark phase differences observed in WT animals likely reflect the combined influence of phase-dependent regulation and photic masking, which act in the same direction under the applied illumination^62^. This is particularly relevant for the SPT and BPT, where external light conditions were aligned with the respective phase, potentially reinforcing behavioral differences. The reduced behavioral amplitude in SNCA animals in their active phase may therefore result from impairments in phase-dependent regulation, photic masking, or both, as these processes share overlapping neural pathways from the retina to hypothalamic structures. For the LDB, however, the coexistence of illuminated and dark compartments introduces potentially opposing influences of photic masking and internal phase-dependent regulation. As a result, behavioral outcomes in this task may reflect a more complex interaction of these mechanisms. Given the experimental design, it is not possible to disentangle the relative contributions of photic masking and phase-dependent processes, which represents a limitation of the present study.

Given the highly fine-tuned nature of light–dark phase-dependent behavior regulation, an important advantage of genetic PD models is the chronic-progressive pathology with ongoing accumulation of *α*-synuclein, similar to the human situation. In two different genetic mouse models, namely the Thy1-*α*-synuclein mouse and the A53T-mutant *α*-synuclein mouse, significant *α*-synuclein deposition within the SCN has already been demonstrated^25,27^. Also, enhanced system-wide neuroinflammation has previously been reported in the SNCA rat model^68^, and it is well established that *α*-synuclein accumulation can trigger microglial activation in vivo and in vitro^69–71^. Supporting this notion, in the Thy1-*α*-synuclein-expressing mouse model, we also demonstrated that *α*-synuclein accumulation in multiple areas of the brain induced neuroinflammation^23^. In human patients as well, multiple lines of evidence suggest that *α*-synuclein accumulation and neuroinflammation play a decisive role in PD pathology. While Lewy pathology has been detected in the SCN of PD patients, such findings are confined to advanced disease stages, with, for example, a median disease duration of fourteen years in one study^16^. Beyond the increased number of activated microglia in the SN^72,73^, elevated concentrations of pro-inflammatory cytokines have been detected in brain tissue^74,75^, cerebrospinal fluid^76^, serum^77,78^, and plasma of patients^79^. Particularly noteworthy is that some of these studies also reported correlations between elevated cytokine levels and non-motor symptoms^77,79,80^. Consistent with this, we observed a specific enrichment of *α*-synuclein in the SCN of SNCA rats. Notably, this accumulation positively correlated with an increased presence of microglial cells. Taken together, we confirmed α-synuclein deposition within the SCN of a rat model of early PD, and present microgliosis as a signature for ongoing neuroinflammation, which could potentially disrupt neuronal circuitry.

In addition to *α*-synuclein accumulation and the increased prevalence of Iba1^+^ microglia, our histological analyses revealed a general reduction in total cell counts within the SCN of SNCA rats. A possible explanation could be the selective loss of specific cellular subpopulations. An alternative, though more speculative explanation, might involve altered neurodevelopment. Impaired neurogenesis has been described in PD patients^81,82^ as well as in the hippocampus of SNCA rats^29^, raising the possibility that developmental or neurogenic deficits could contribute to SCN alterations. However, given the nature of our findings, enhanced neurodegeneration appears to be the more likely explanation. Importantly, our data further demonstrated that neither the total number nor the relative proportion of VP and/or VIP^+^ cells was altered in this model. VIP and VP represent the two major neuropeptidergic subpopulations within the SCN and play complementary roles in circadian timekeeping, with VIP neurons primarily mediating synchronization of cellular oscillators^31,32^ and VP neurons contributing to the generation and stability of circadian output signals^33^. As these two subpopulations remained unchanged, the observed light-dark phase-dependent effects are unlikely to be attributable to alterations in VP- or VIP-mediated signaling.

In contrast, we found increased proportional representation of Orexin A^+^ fibers and NeuN^+^ neurons within the SCN of SNCA rats. Orexin A^+^ neurons project into the SCN, where they modulate light–dark phase-dependent rhythm regulation^34–36,83^. In an MPTP-induced mouse model of PD, Orexin A was shown to exert a neuroprotective effect on dopaminergic neurons in the SN^84^. Furthermore, the orexinergic system has been implicated in sensory-motor deficits associated with PD^85^. In human studies, a dramatic reduction—up to 62% fewer—of Orexin A^+^ neurons in the hypothalamus of PD patients was revealed, and correlated with disease stage^86,87^. In light of this, the increased density of Orexin A^+^ fibers we observed in young SNCA rats is unlikely to represent a protective mechanism. Rather, it may reflect an early state of orexinergic hyperactivity or abnormal input from the hypothalamus, potentially driven by local *α*-synuclein pathology. Such an overactive state could transiently enhance orexinergic signaling within the SCN, thereby perturbing light–dark phase-dependent regulation. This interpretation would also align with the later loss of orexinergic neurons observed in human PD, suggesting a shift from early dysregulation toward eventual degeneration.

Our study is limited by relatively small cohort sizes, although this is in line with other recent work in the field^23,29,88^. We also focused exclusively on male animals, as female SNCA rats do not develop significant PD symptoms even at older ages^37^, leaving the role of sex-specific differences to be addressed in future studies. A further limitation of the present study is that we did not perform continuous long-term circadian monitoring, such as recordings of rest-activity cycles, core body temperature, or sleep-wake cycles via electroencephalography (EEG), nor did we assess rhythmicity under constant environmental conditions (e.g., constant darkness or constant light). Such approaches would be required to reliably characterize endogenous circadian function and its potential disruption. Therefore, our data do not allow direct conclusions about intrinsic circadian rhythm alterations, but are limited to phase-dependent behavioral differences under standard light–dark conditions. Another limitation is that we did not conduct a dedicated vision test to rule out visual impairments. The surface pellet test revealed no genotype differences within a given light–dark phase, indicating preserved basic visual function under our conditions. Moreover, given the young age of our animals (3 months), visual dysfunction is not expected at this stage. However, previous studies have linked *α*-synuclein pathology to visual deficits following intravitreal injection of *α*-synuclein fibrils in mice^89^. Although this approach differs from a genetic model, it suggests that *α*-synuclein pathology may potentially affect light-mediated signaling through ipRGCs and influence photic masking. Consequently, we cannot fully exclude that *α*-synuclein in our SNCA rats may influence the transmission of light signals to the brain and within the central nervous system. Future work should also include a systematic analysis of sleep–wake cycles and sleep architecture in this model.

Taken together, our findings suggest that light–dark phase-dependent behavior dysfunction emerges already at very early stages of PD pathology in our animal model, in the absence of overt dopaminergic neurodegeneration. By linking *α*-synuclein accumulation in the SCN to local neuroinflammatory processes and alterations in orexinergic input, our study highlights novel mechanisms that may underlie day/night rhythm disturbances in PD. These results not only provide a framework for understanding how early molecular and cellular changes translate into behavioral phenotypes but also suggest potential targets for therapeutic intervention aimed at stabilizing non-motor symptoms during the early and potentially also prodromal phases of the disease.

## Methods

### Animals

All animal procedures complied with the European Directive 2010/63/EU and the ARRIVE guidelines and were approved by the local authority for animal welfare (Landesamt für Landwirtschaft, Lebensmittelsicherheit und Fischerei Mecklenburg-Vorpommern, Germany: M-V/7221.3-1-002/21). Male Sprague-Dawley rats aged three months (310–490 g) were used for all experiments, as females of this age were shown to be asymptomatic^37^. Animals were bred in the central animal facility of the University Medical Center Rostock, either as wild-types or as homozygous SNCA rats overexpressing the full-length human *SNCA* gene under the control of its native regulatory elements, generated via heterozygous pairings (SPRD-Tg(RP11-115D19)2Uhg). Age- and sex-matched wild-type littermates served as controls. Animals were transferred to the experimental facility at the age of 2 months. Rats were group-housed under standardized environmental conditions with controlled temperature and humidity and maintained on a 12-h light–dark cycle (lights on 6 a.m. with 250 lx and lights off 6 p.m.). Food and water were available *ad libitum*. A total of 27 rats were included in the final analysis (inactive groups: N_WT_ = 6, N_SNCA_ = 6; active groups: N_WT_ = 9, N_SNCA_ = 6).

### Genotyping

To distinguish homozygous and heterozygous SNCA transgenic rats from their WT littermates for both breeding and experimental allocation, genotyping was performed. DNA was extracted from a small ear tissue sample collected during animal labeling. Samples were incubated overnight at 56 °C in a custom-prepared lysis buffer containing 100 mM Tris-HCl (pH 8.5), 5 mM EDTA (pH 7.4), 0.2% sodium dodecyl sulfate (SDS), 200 mM NaCl, and 20 mg/ml proteinase K. DNA isolation and subsequent quantitative polymerase chain reaction (qPCR) followed the procedure established by Nuber et al.^28^.

Briefly, DNA (20 ng/μl) was combined with 100 μM of each primer (Eurofins Genomics, Germany): SNCA-F (5′-TGT TTG CAC ACA CCA GGA TG-3′), SNCA-R (5′-ATT GCG GAG GGA AGA GGA AA-3′); *β*-Actin-F (5′-AGC CAT GTA CGT AGC CAT CCA-3′), and *β*-Actin-R (5′-TCT CCG GAG TCC ATC ACA ATG-3′). For detection, 1 μM hybridization probes were used: SNCA (5′-TGC CCT TTC TAC CTG GTT GA-3′) and *β*-Actin (5′-TGT CCC TGT ATG CCT CTG GTC GTA CCA C-3′). qPCR amplification was carried out with the following thermal profile: 2 min at 95 °C, followed by 43 cycles of 15 s at 95 °C and 1 min at 56 °C. All reactions were run in duplicate and normalized to *β*-Actin expression.

### Behavioral tests

All animals underwent a one-week acclimatization period prior to the onset of behavioral testing to allow recovery from potential transport-related stress and to adapt to the housing environment. To assess behavioral performance under different light–dark conditions, animals of both genotypes (WT and SNCA) were evenly distributed into two experimental cohorts: active and inactive. All behavioral testing was performed under the lighting conditions defined by the 12:12 light–dark cycle. The inactive group was assessed during the early light phase under standard illumination (250 lx) in a quiet and controlled setting. The active group was handled during the initial hours of their dark phase under dim red-light conditions, which are not visible to rodents, to minimize external disturbances. The testing sessions started one hour after lights-off and were conducted under dim red light. The LDB Test was the only exception, as visible light was required; illumination was restricted exclusively to the interior of the light compartment, while the testing room remained in darkness. No other nighttime behavioral task involved exposure to visible light. The behavioral data of WT animals under both lighting conditions (active and inactive) have been previously published in Weber et al. (2024) and were re-analyzed here together with the SNCA cohorts for direct comparison^90^.

**Fig. 4:**
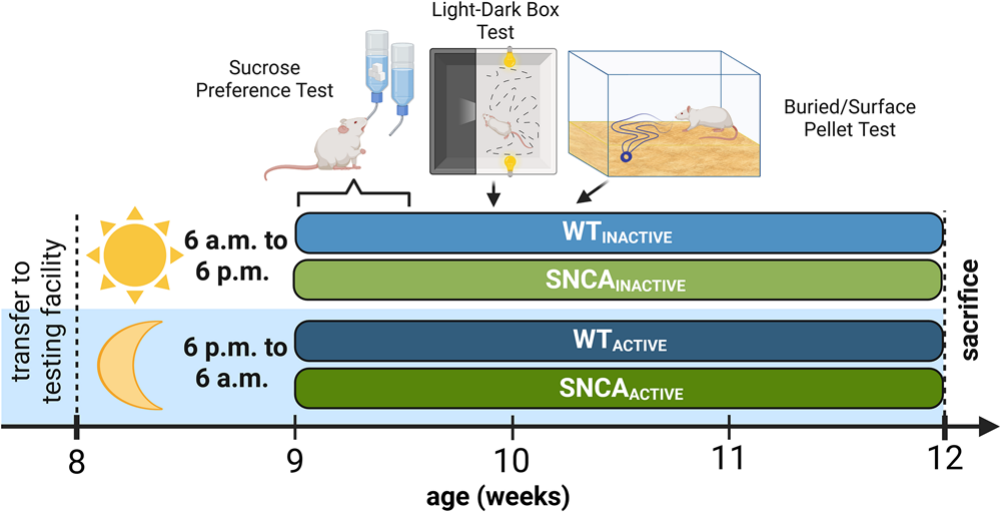
Experimental setup and timeline of behavioral experiments. For experiments, animals were around two months old at the beginning of behavioral tests. Both wild-type (WT) and *α*-synuclein overexpressing rats (SNCA) underwent the same set of behavioral testing, including the sucrose preference test, the light-dark box test, and the buried/surface pellet test. Zeitgeber time (ZT) was defined with ZT_0_ corresponding to lights on and ZT_12_ to lights off. Each behavior test was either conducted during the inactive phase (ZT_0_ to ZT_12_ with lights on, depicted by a sun) or the active phase of the animals (ZT_12_ to ZT_24_ with lights off, depicted by a moon). Created in BioRender. Weber, H. (2025) https://BioRender.com/evb544j.

Zeitgeber time (ZT) was defined with ZT_0_ corresponding to lights on at 6 a.m. and ZT_12_ to lights off at 6 p.m. local time. For behavioral testing, animals were relocated to the respective testing facility either at ZT_0_ (inactive cohort) or ZT_12_ (active cohort), followed by a 1-h habituation period prior to testing. A set of three behavioral paradigms was employed to comprehensively evaluate various behavioral domains. To prevent carry-over effects, a minimum recovery period of two days was maintained between tests. A schematic overview of the experimental timeline is provided in Fig. 4.

The sucrose preference test (SPT) is a well-established method for assessing (an)hedonic behavior or stress-related phenotypes in rodents^91^. To familiarize the animals with the choice paradigm, all rats were given continuous access to two bottles containing plain water 2 days prior to and throughout the testing period^92^. One hour before each session (inactive cohort; ZT_0_ to ZT_1_, active cohort; ZT_12_ to ZT_13_), rats were individually housed in new cages and again provided with two plain water bottles. Immediately before the behavioral assessment, freshly prepared bottles containing either plain water or a 2% sucrose solution were weighed and placed side by side in the cages^93^. After a 4-h test session (inactive cohort; ZT_1_ to ZT_5_, active cohort; ZT_13_ to ZT_17_), the bottles were reweighed and subsequently cleaned. This procedure was repeated on four consecutive days, with daily alternation of the left–right bottle positions to control for side preference^94^. For each animal, the mean values of the relative sucrose consumption (total sucrose consumption divided by total consumption multiplied by 100) over the 4 test days were calculated.

The light–dark box (LDB) test is a widely established paradigm to assess anxiety-related and exploratory behavior in rodents^95^. The custom-built acrylic LDB apparatus consisted of two compartments: an illuminated area (34 cm × 51 cm, 65 lx) and a dark area (17 cm × 51 cm, 0 lx), separated by an opaque divider with a central opening (8 cm × 8 cm)^96^.

Rats were transferred an hour prior to the actual testing into the testing room (inactive cohort; ZT_0_ to ZT_1_, active cohort; ZT_12_ to ZT_13_). Each testing session lasted approximately 3–4 h, with individual trials conducted sequentially (inactive cohort; ZT_1_ to ZT_5_, active cohort; ZT_13_ to ZT_17_). To minimize neophobic responses, the box was pre-marked by male rats not involved in the study. Between trials, the apparatus was wiped with wet towels to preserve odor cues while maintaining hygiene^97^. Test subjects were placed individually into the center of the lit compartment, facing away from the doorway. Each trial lasted 10 minutes, during which several parameters were recorded: latency to first enter the dark compartment, total time spent in the lit area, number of transitions between compartments, frequency of rearing behaviors, and total distance traveled (TDT). Behavioral data were video-recorded and analyzed using EthoVision XT 8.5 software (Noldus, Wageningen, Netherlands). One animal displayed conspicuously deviant behavior in the LDB test. For two of the five measured parameters, their values were identified as statistical outliers. To ensure consistency of statistical analysis across all readouts, this animal was excluded from the LDB dataset.

The buried pellet test (BPT) is a standard procedure used to evaluate olfactory function in rodents by assessing their ability to detect and retrieve a hidden food item using scent cues^98^. To familiarize the animals with the test stimulus and avoid neophobic responses, each rat received five to ten Froot Loops (Kellogg’s®, Kellanova, USA) per day over the two days preceding the experiment.

Food restriction was initiated ten hours prior ZT_1_ or ZT_13_ to increase motivation for food retrieval^99^. Again, animals were transferred within their home cages to the experimental room at ZT_0_ or ZT_12_ to the testing room for habituation.

For testing, each subject was placed in a fresh type III cage containing 6 cm of clean bedding. A single Froot Loop was buried approximately 4 cm beneath the bedding surface, and the animal was introduced at the opposite end of the cage. The latency to locate and retrieve the pellet was recorded. Each BPT session lasted approximately 2–3 h, with individual trials conducted sequentially (inactive cohort; ZT_1_ to ZT_3,5_, active cohort; ZT_13_ to ZT_15,5_).

Following completion of the BPT for all animals, a Surface Pellet Test (SFPT) was conducted using a comparable setup. Each SFPT session lasted approximately 1-2 hours (inactive cohort; ZT_3,5_ to ZT_5_, active cohort; ZT_15,5_ to ZT_17_). In contrast to the BPT, the Froot Loop was placed visibly on the bedding surface. The positions of both the pellet and the rat were systematically altered but kept consistent across subjects^100^.

### Transcardial perfusion

At the age of three months, rats were deeply anesthetized using isoflurane, and subsequently received an intraperitoneal, weight-adjusted injection of a mixture containing ketamine (10% Ketanest, 25 mg/ml) and xylazine (2% Rompun, 1.25 mg/ml) dissolved in 0.9% saline. Transcardial perfusion was performed with ice-cold 4% paraformaldehyde (PFA) in 0.1 M phosphate buffer (pH 7.4). Brains were subsequently prepared for histological analyses. All rats were sacrificed in the first hours of their light phase. Extracted brains were post-fixed in 4% PFA (0.1 M phosphate buffer, pH 7.4) for 24 h at 4 °C and then cryoprotected by immersion in 30% sucrose in phosphate buffer for 48 h at 4 °C. After cryoprotection, the tissue was rapidly frozen and stored at −80 °C. For staining procedures, brains were sectioned coronally at 30 μm thickness and preserved in a cryoprotectant solution at −20 °C until further use.

### Tyrosine hydroxylase diaminobenzidine (TH-DAB) staining

To quantify potential dopaminergic cell loss in the *substantia nigra* (SN) and the ventral tegmental area (VTA), TH immunostaining was carried out as described in Brandt et al.^101^. Briefly, free-floating coronal brain sections were incubated with a mouse primary anti-TH antibody (1:1000; Sigma Aldrich, US, RRID: AB_477560). Detection was performed using the ABC Elite Kit (Vector Laboratories, US, RRID: AB_2336827), which provides the biotinylated secondary antibody together with the ABC and DAB components. TH DAB-stained images were acquired in bright-field mode using an Axio Imager.A1 microscope (Carl Zeiss, Oberkochen, Germany) with a ×20 objective.

### Immunofluorescence staining

To investigate protein expression in the SCN, two triple-labeling approaches were applied: the first combination included the rat anti-human *α*-synuclein (1:50; Enzo Life Science, US, RRID: AB_2050691), guinea pig anti-Orexin A (1:500; Synaptic System, Germany, RRID: AB_2713976), and rabbit anti-Iba1 (1:500; US, Germany, RRID: AB_839504), while the second targeted rabbit anti-vasopressin (VP) (1:500; Abcam; UK, RRID: AB_3099753), chicken anti-NeuN (1:1,000; MilliporeSigma; US, RRID: AB_11205760), and mouse anti-vasoactive intestinal peptide (VIP) (1:200; ThermoFisher; US, RRID: AB_2877573). Coronal cryosections were processed according to standard immunofluorescence protocols. Briefly, sections were incubated for 30 min in citrate buffer at 95 °C, followed by blocking in 8% donkey serum and 2% Triton X-100 in TBST for 1 h at room temperature. Subsequently, sections were incubated overnight at 4 °C with primary antibodies, and the following day with the corresponding fluorescence-conjugated secondary antibodies for 2 h at room temperature. Nuclei were counterstained with Hoechst33342 for 3 min at room temperature. For a negative control, all processing steps were identically repeated to this staining protocol, except that primary antibodies were omitted during the overnight incubation, and sections were incubated only with the corresponding secondary antibodies. Under these conditions, no relevant signal was detected in either genotype. Representative images of the negative control stainings are provided in the Supplementary Fig. S1.

Immunofluorescence images were captured using an Axio Observer Z1 microscope with a ×20 objective, employing ZEN Blue software and the Tiles and Position module (Carl Zeiss). Due to the very small size of the SCN, it was not possible to obtain both staining combinations from every animal. As a result, not all animals contributed data points to each analysis.

### Image processing and quantification

Image analyses were performed using ZEN Blue software from Zeiss with its analysis module. Cell quantification was carried out automatically, with the exception of Orexin A-positive fibers, which were counted manually. For automated analysis, eight to fourteen z-stacks (1 μm spacing) were acquired and merged using the “Extended Depth of Focus” with the “Maximum Projection” function to generate a single high-contrast composite image per region. This merged image was then subjected to automated quantification of cells. The detailed settings used for each automated analysis are provided in the Supplementary Table 1. As the extended depth projection integrates multiple optical sections, cell counts were normalized to the corresponding analyzed volume in the later analysis. To allow comparison across animals, the proportion of positive VIP, VP, or Iba1 cells and Orexin fibers is reported relative to the total number of SCN nuclei, as determined by Hoechst33342 counterstaining, thus representing the percentage of SCN cells expressing the target marker.

For *α*-synuclein and TH^+^ fibers in the dorsal and ventral striatum, expression levels were quantified by measuring fluorescence intensity within the SCN and subtracting background intensity values obtained from the adjacent optic chiasm or the striatum with background values from the anterior commissure (named hereafter “corrected” intensity).

### Statistical analysis

Data were analyzed and visualized using GraphPad Prism 10.6.0 (GraphPad Software Inc.), R Studio 2025.09.0 (Posit Software), and Inkscape 1.3. Potential outliers were identified using Grubb’s test with a 0.5% quantile. For behavioral experiments, two-way ANOVAs were performed to assess the influence of genotype and daytime, followed by Bonferroni’s correction for multiple comparisons. For histological analyses, group differences were evaluated using unpaired Student’s *t* tests or Welch’s *t* tests, as appropriate. Results are reported as mean ± standard error of the mean. In addition, correlations between histological and behavioral measures were assessed using Pearson’s correlation coefficient. Statistical significance was set at *p* < 0.05. All data are presented in boxplots, with the central line indicating the median, box edges corresponding to the 25th and 75th percentiles, and whiskers denoting the minimum and maximum values, to show a full representation of the data. Each dot represents an individual data point, with outliers displayed as white dots. Unless stated otherwise, results are reported as mean ± standard error of the mean.

## Supplementary information

Supplementary Information

## Data Availability

All data generated and analyzed during this study have been deposited in the Zenodo repository and are publicly available at 10.5281/zenodo.20266946 (https://zenodo.org/records/20266947).
