# Supplementary material for: Altered light-dark phase-dependent behavioral responses and suprachiasmatic nucleus pathology in an α-synuclein rat model of Parkinson’s disease
Source: NPJ Parkinsons Dis. 2026 Jun 19;12:153. doi: 10.1038/s41531-026-01436-2 (PMC13282383; doi:10.1038/s41531-026-01436-2)
Supplement: Supplementary file 1 — Supplementary Information [file 41531_2026_1436_MOESM1_ESM.pdf]

Supplementary Information

Altered light-dark phase-dependent behavioral responses and suprachiasmatic nucleus pathology in an α-synuclein rat model of Parkinson's disease

Hanna Weber, Meike Statz, Nicolas Casadei, Olaf Riess, Franziska Richter, Wiebke Hermann, Alexander Storch, Mareike Fauser

List of Supplementary Informations

Supplementary Table 1: Settings for automated cell counting using ZEN Blue software from Zeiss. The table summarizes the parameters applied for automated detection of SCN cells.

Supplementary Figure S1: Negative control stainings performed with only the respective secondary antibody of the conducted histological analysis of the suprachiasmatic nucleus (SCN) in wild-type (WT) and α-synuclein overexpressing rats (SNCA rats).

Supplementary Figure S2: Tyrosine hydroxylase (TH) DAB-Staining in the *substantia nigra* (SN) and ventral tegmental area (VTA) of wild-type (WT) and α-synuclein expressing rats (SNCA).

Supplementary Figure S3: Heatmap showing Pearson correlation coefficients between corrected α-synuclein immunoreactivity and cellular parameters in the suprachiasmatic nucleus (SCN).

Target	Channel	Smoothing	Sigma	Sharpen	Pick Be-havior	Tol-erance	Neight-boor-hood	Min. Area	Min. Hole Area	Fill Holes	Binary	Count	Seperate	Count	Supress Invalid
Hoechst	H3342	Gauss	0.5	None	+	3%	0	30	1	No	Open	3	Watersheds	3	No
VIP	488	None	-	None	+	3%	0	47	1	Yes	None	-	Morphology	20	No
VP	555	None	-	None	+	3%	1	52	1	Yes	Erode	3	Morphology	3	No
NeuN	647	Median	3	None	+	3%	0	200	1	No	Open	3	Watersheds	3	No
Iba1	647	Median	3	None	+	3%	0	500	1	No	Open	3	Morphology	3	No

Supplementary Table 1: **Settings for automated cell counting using ZEN Blue software from Zeiss. The table summarizes the parameters applied for automated detection of SCN cells.** For each image (captured with 20-fold magnification), the histogram's high filter was set to the maximum value, and the low filter was individually adjusted until the software clearly identified positive cells. All other parameters were held constant across all images to ensure comparability.

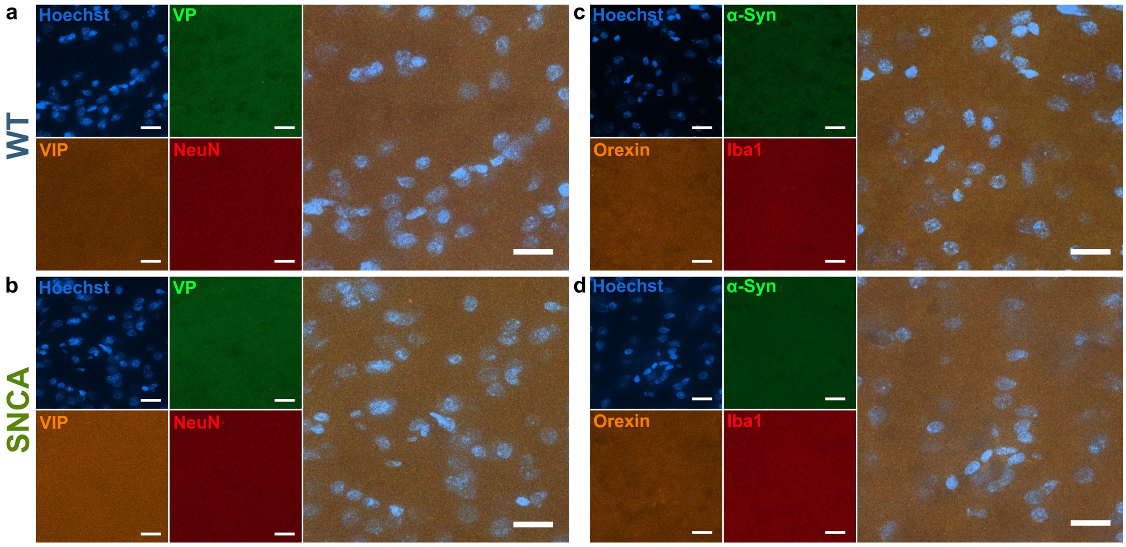

Supplementary Figure S 1: Negative control stainings performed with only the respective secondary antibody of the conducted histological analysis of the suprachiasmatic nucleus (SCN) in wild-type (WT) and α-synuclein overexpressing rats (SNCA rats). (a, b) Representative negative control of the vasopressin (VP), vasoactive intestinal peptide (VIP), and NeuN staining of the SCN of WT (a) and SNCA (b) rats. Scale bar = 20 µm. **(c,d)** Representative negative control of the α-synuclein, Orexin and Iba 1 staining of the SCN of WT (c) and SNCA (d) rats. Scale bar = 20 µm.

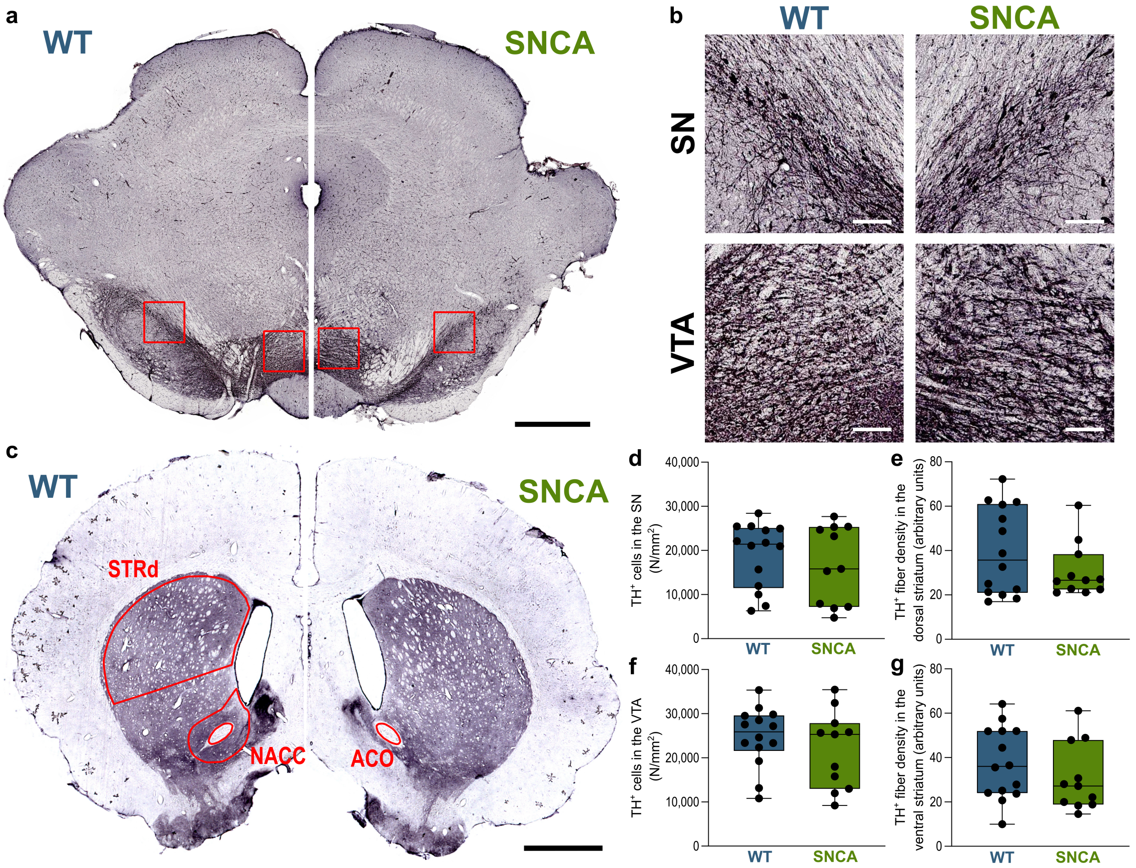

Supplementary Figure S 2: Tyrosine hydroxylase (TH) DAB-Staining in the *substantia nigra* (SN) and ventral tegmental area (VTA) of wild-type (WT) and α-synuclein expressing rats (SNCA). (a) Representative TH stainings of the SN and VTA of WT and SNCA rats. Scale bar = 1,000 µm. **(b)** Magnifications of representative TH stainings of the SN and VTA of WT and SNCA rats. Scale bar = 100 µm. **(a)** Representative TH stainings of the dorsal striatum (STRd), *Nucleus accumbens core* region (NACC), and the anterior commissure (ACO). Scale bar = 1,000 µM. **(d)** Quantification of TH-positive neurons in the SN. No significant differences were observed between groups. **(e)** Quantification of TH-positive neurons in the VTA without significant group differences. **(f)** Quantification of TH-positive fibers in the dorsal striatum did not reveal any group differences. **(g)** Quantification of TH-positive fibers in the ventral striatum. No significant differences were detected between groups. Data are presented as boxplots with median (central line), interquartile range (box), and minimum/maximum values (whiskers). Individual data points are shown as dots.

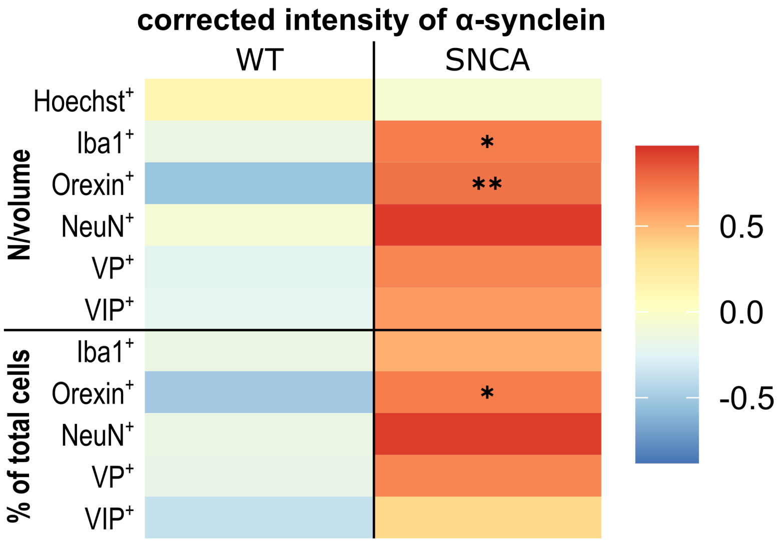

Supplementary Figure S 3: Heatmap showing Pearson correlation coefficients between corrected α-synuclein immunoreactivity and cellular measures in the suprachiasmatic nucleus (SCN). Columns represent wild-type (WT; left) and α-synuclein expressing rats (SNCA; right), and rows depict cell or fiber counts (N/mm^3) for Hoechst, Iba1, Orexin A, NeuN, vasopressin (VP), and vasoactive intestinal peptide (VIP) cells/fibers (top), as well as percentages relative to total Hoechst$^+$ cells for Iba1, Orexin A, NeuN, VP, and VIP (bottom). In WT rats, correlations are negligible due to the absence of detectable α-synuclein, whereas SNCA rats show uniformly positive correlations, with significant correlations between α-synuclein intensity and Iba1$^+$ cells and Orexin A$^+$ fibers, respectively. Color intensity represents the magnitude of the correlation coefficient (Pearson r; $^*p < 0.05$, $^{**}p < 0.01$).
